# A fast hyperspectral change detection algorithm for agricultural crops based on spatial reconstruction

**DOI:** 10.1371/journal.pone.0323446

**Published:** 2025-05-15

**Authors:** Jianghong Yuan, Er-Yang Chen, Haiyin Qing

**Affiliations:** 1 Sichuan Railway College, Chengdu, China; 2 Education Department of Sichuan Province, Key Laboratory of Detection and Application of Space Effect in Southwest Sichuan at Leshan Normal University, Leshan, China; 3 School of Electronic Information and Electrical Engineering, Chengdu University, Chengdu, China; 4 Geomathematics Key Laboratory of Sichuan Province, Chengdu University of Technology, Chengdu, China; Indian Institute of Technology Jammu, INDIA

## Abstract

Crop change detection plays a pivotal role in ensuring agricultural sustainability and environmental monitoring. Leveraging the high spectral resolution of hyperspectral imagery and bi-temporal analysis, this study presents a Fast Hyperspectral Change Detection algorithm based on Spatial Reconstruction (FHCDSR) designed to identify subtle agricultural changes with improved accuracy and computational efficiency. The proposed method incorporates three key innovations: (1) boundary-constrained preprocessing of 3D hyperspectral data, (2) Laplacian-regularized spatial reconstruction, and (3) a novel tensor-based change detection framework. We conduct a comprehensive evaluation of FHCDSR using two datasets: the Hermiston dataset and the Yancheng dataset. Experimental results demonstrate that FHCDSR achieves superior performance on both datasets, with AUC values of 90.20% (Hermiston) and 95.39% (Yancheng), outperforming six state-of-the-art comparison methods by 3.39–14.78% in detection accuracy. Remarkably, the algorithm maintains high computational efficiency, completing analyses in 9.76 seconds (Hermiston) and 10.90 seconds (Yancheng), representing up to 94.05% reduction in processing time compared to conventional methods. The consistent performance across different agricultural landscapes highlights FHCDSR’s robustness as an unsupervised change detection solution, with significant potential for precision agriculture and wetland ecosystem monitoring applications.

## Introduction

Sustainable agricultural productivity and food security are two of the paramount global challenges of the 21st century [1]. Crop change detection research serves as the cornerstone of agricultural sustainability, playing a pivotal role in bolstering the resilience of agricultural systems, mitigating production risks, and enhancing the welfare of rural communities, thereby safeguarding the sustainability of food supply. Change detection is one of the key applications of remote sensing technology, which is crucial in the continuous monitoring and identification of changes in image scenes [2–4] has been widely used in crop change detection, environmental monitoring, land change analysis, urban expansion assessment, disaster detection and assessment, and military battlefield monitoring, and has achieved remarkable results [5–8].

In recent years, as China, the United States, Germany, Japan and other major economies in the world pay more and more attention to “low-altitude economy”, people’s demand for “low-altitude economy” related technologies is also increasing. Hyperspectral/multispectral change detection, as an important technical branch, has gradually be-come a new research hotspot. Hyperspectral images (HSIs) differ significantly from multi-temporal color or high-resolution remote sensing images [9] in their organizational structure, presenting data in three-dimensional (3D) cubes that incorporate both spatial and spectral dimensions. This structure enables HSIs to offer intricate spectral “diagnostic” information [10], making them a valuable tool for various applications. The wealth of data information, combined with the inherent strengths of remote sensing images, including their extensive coverage and rapid detection cycles [11,12], confers significant potential to hyperspectral change detection in accurately identifying crop changes and diverse agricultural transitions [13]. Therefore, the timely and precise acquisition of crop seeding change information is crucial for informing national or regional agricultural economic strategies, guiding planting structure adjustments, and enhancing the management of agricultural production.

Over the past decade, various techniques have been developed for change detection using hyperspectral images (HSIs) across different fields of application. Upon comprehensive review and analysis, HSI change detection can generally be categorized into four broad groupings [14–16]:

1) Algebraic methodologies primarily involve techniques such as image differencing, image ratioing, image regression, Absolute Distance (AD) [17], Absolute Average Difference (AAD) [18], among others. These methods utilize direct algebraic operations on bi-temporal Hyperspectral Images (HSIs) to detect pixel-level changes. They are lauded for their simplicity and computational speed. However, they operate under the premise that changes are directly reflected in the variation of pixel intensity values, which may not always hold true.
2) Transformation-oriented techniques, including Conventional Principal Component Analysis (CPCA), Temporal Principal Component Analysis (TPCA) [19], sub-space-based change detection (SCD) [20], local SCD (LSCD) [20], and related methodologies. These techniques transform data into new feature spaces to detect changes, yet they might not capture the subtleties of continuous spectral variations or the nuanced relationships between pixels[21].
3) Classification-based strategies [14,22–24] can be divided into post-classification or direct classification of bi-temporal imagery. Post-classification involves processing images from different time periods separately to account for environmental variations. Direct classification, on the other hand, integrates multitemporal data for a holistic analysis, identifying changes across categories. These methods, however, require sophisticated classification algorithms to be effective.
4) Advanced methodologies include Unmixing-based [25], Low Rank and Sparse Representation-based [15,26], Deep Learning-based [13,27]-[28], Tensor-based [29–30], and similar techniques[4,11–12]. Deep learning approaches are particularly adept at extracting advanced features through data-driven transformations, with their success contingent upon the size and precision of the training datasets.

Despite the commendable effectiveness of the aforementioned algorithms across various disciplines [31,32], their practical deployment is still confronted with significant challenges. This is due to the absence of tailored algorithms specifically designed for crop change detection. Some are unable to efficiently process the spatial relationships between different crop pixels, thereby limiting detection accuracy. Others rely heavily on extensive training data to ensure precision, which in turn increases the time cost.

Spatial-spectral information plays a pivotal role in enhancing change detection precision [33]; exploiting it involves not merely extracting spatial details but delving into the intrinsic spectral characteristics to achieve superior results [34,35]. Against this backdrop, this work introduces a novel Fast Crop Hyperspectral Change Detection via Spatial Re-construction (FHCDSR) approach.

The FHCDSR methodology commences by acknowledging the inherent similarities among different crop spectra, thereby amalgamating spectral information to accentuate the boundary delineations of distinct ground objects within hyperspectral imagery, culminating in spatial reconstruction of two-phase crop hyperspectral data. Subsequently, a purpose-built detector is employed to augment the accuracy of detecting bi-temporal crop changes. The FHCDSR method has shown excellent performance on the Hermiston dataset and the Yancheng dataset, eliminating the dependence on prior knowledge and sample data in agricultural change detection. It seamlessly blends spectral information, refines the boundaries of different crops, and exploits the full potential of spatial morphological features, thereby improving the performance of change detection techniques. The algorithm parameters are simple, which greatly improves operational efficiency.

FHCDSR addresses agricultural monitoring challenges through its Laplacian-constrained spatial reconstruction, reducing sensitivity to weather-induced noise.The key contributions of this research can be encapsulated as follows:

1. FHCDSR is a novel unsupervised change detection method that eliminates the reliance on prior knowledge and sample data in the context of agricultural change detection.
2. The method harmoniously blends spectral information, accentuates the boundary lines of various crops, and harnesses the full potential of spatial morphological features. It deftly resolves the issue of detection inefficiency stemming from irregular spectral pixel interference. As a result, this enhancement substantially elevates the performance of change detection techniques.
3. Considering the inherent characteristics of hyperspectral data, this method can better separate the variable pixels and the invariant pixels, and the algorithm parameters are simple, which significantly improves the operation efficiency of the algorithm.

The structure of the paper is as follows: Section 2 details the materials and methods. Section 3 presents the experiment and analysis with the Hermiston dataset and the Yancheng dataset. Finally, in section 4, the whole research is summarized.

## Materials and methods

### A.Materials

**Fig 1:**
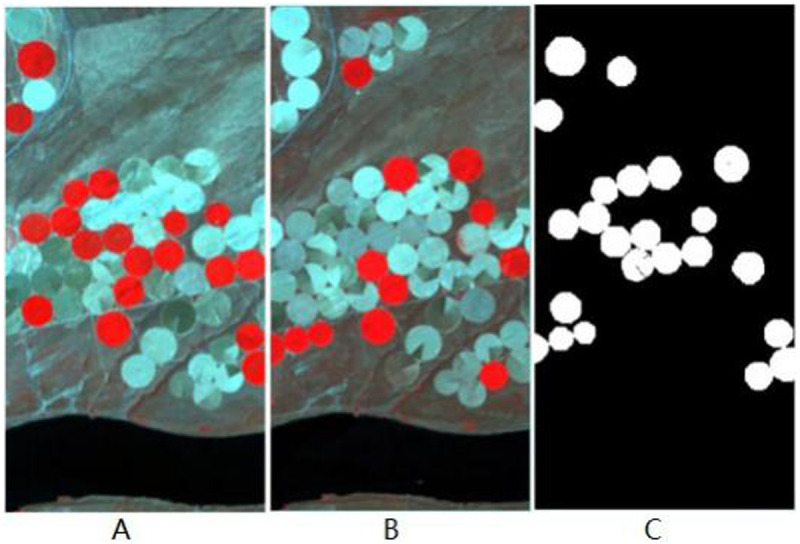
Illustration of the Hermiston dataset. (A) Farmland on 1 May 2004, (B) Farmland on 8 May 2007, (C) Ground-truth change map.

The first dataset is the Hermiston dataset, which encompasses farmland and river regions and is located in Hermiston, Oregon, United States. These hyperspectral images were taken in 2004 and 2007 using the Hyperion sensor, and these hyperspectral images contain a total of 242 spectral bands, each of which has the size of 390 × 200 pixels. Collectively, these images comprise a total of 242 bands. However, bands B001-B007, B058-B076, and B225-B242 remain un-calibrated, thus necessitating their exclusion from our processing, which is a preprocessing step. Within this scene, five distinct types of changes pertaining to crop transitions have been pinpointed. Additionally, the complete Hermiston image is meticulously la-belled with 78,000 pixels, comprising 9986 positive pixels and 68,014 negative pixels [36]. The detailed images and the ground-truth map are illustrated in Fig 1.

The second dataset is the Yancheng dataset, comprising two bi-temporal images captured from a wetland agricultural area in Yancheng city, Jiangsu Province, China on May 3, 2006 and April 23, 2007. After eliminating noisy and water absorption bands, the remaining scene, sized 400 × 145 pixels with 154 spectral bands, was utilized. The primary change type is also farmland land - cover change.

### B.Methods

**Fig 2:**
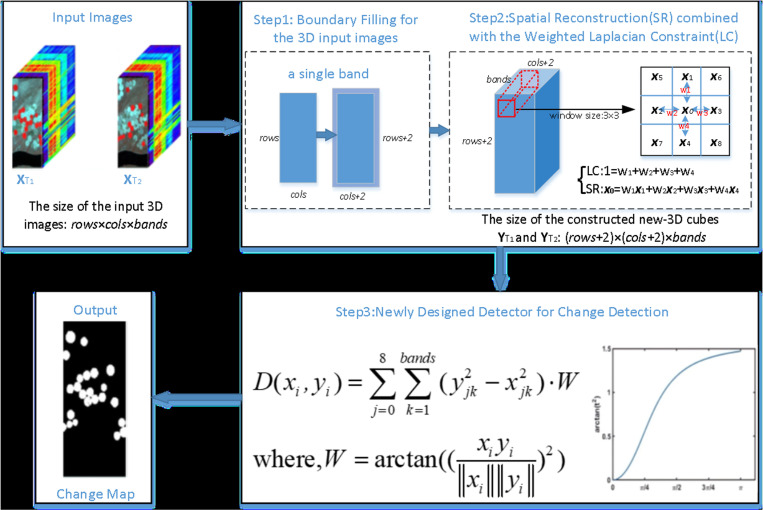
Flowchart of the method.

The workflow of the FHCDSR method is illustrated (Fig 2), comprising three steps: firstly, the boundary filling for the preprocessed 3D input images; secondly, Spatial Reconstruction combined with the Laplacian Constraint; and finally, a newly designed detector for change detection.

#### 1) Boundary filling.

During the process of spatial reconstruction and the design phase of the change detector, a 3x3 spatial window is chosen for analysis. To ensure the efficient handling of boundary information within the hyperspectral images, it is imperative to perform boundary filling on the input images prior to processing [37].

Let $i_{r,c}(1\leq r\leq rows,1\leq c\leq cols)$ represent the pixel values corresponding a single band $I_{r,c}$ in ${\left\{I_{r,c,b}\right\}}_{1\leq r\leq rows,1\leq c\leq cols,1\leq b\leq bands}\in R^{rows\times cols\times bands}$, which is called the input 3D cube.

Then, an enlarged-3D cube ${\left\{X_{r,c,b}\right\}}_{1\leq r\leq (rows+2),1\leq c\leq (cols+2),1\leq b\leq bands}\in R^{(rows+2times(cols+2)\times bands}$ can be obtained by boundary filling, and the boundary filling for every single band is performed according to the following rules:

(1) $i_{r,c}(2\leq r\leq (rows+1),2\leq c\leq (cols+1))=x_{r,c}(1\leq r\leq rows,1\leq c\leq cols)$;
(2) for those pixels distributed at the edge of $X_{r,c}$, a mirror image is used to fill in the neighboring blank pixels.

#### 2) Spatial Reconstruction based on Weighted Laplacian Constraint.

In step2, as shown in Fig 1, the local 3D cube of interest contains 9 spectra, which can be represented as:${\left\{X_{i}\right\}}_{0\leq i\leq 8}\in R^{1\times bands}$. Then the distance between the spectrum at the center and its four nearest neighbors can be easily calculated by means of cosine distance, Euclidean distance, spectral Angle distance, etc. These calculated distance values are normalized and called weights.

Based on the weights obtained above, the weighted Laplacian vector can be constructed for the spectral vector $X_{0}$ of interest:


$$\nabla^{2}(X_{0})=w_{0}X_{0}-w_{1}X_{1}-w_{2}X_{2}-w_{3}X_{4}-w_{4}X_{4}$$ (1)

in which, $w_{0}\in R$ is a trade-off parameter.

To incorporate the smoothness across the neighboring pixels, $\nabla^{2}(X_{0})$ can be set to zero [38].

Normally, we can assign,$w_{0}=1$, and $w_{i}(i=1,...,4)$ are normalized as $\overset{\sim }{w_{i}}\;=\frac{w_{i}}{\eta }$, where $\eta =\sum {\mathrm{\backslash nolimits}}_{i=1}^{4}w_{i}$.

After the same processing of the bi-temporal dataset is completed, the reconstructed new 3D datasets $Y_{T_{1}}$ and $Y_{T_{2}}$ corresponding to the scene at time T1 and T2, respectively, are carried out to change detection.

#### 3) Newly designed detector for change detection.

Upon reconstruction of the bi-temporal dataset, the spectral variation in unchanged pixels is diminished. Concurrently, recognizing the significant impact that local background pixels exert on the target pixel, particularly the eight adjacent pixels that exhibit close spectral and spatial similarity, an innovative detection algorithm is introduced to enhance change detection precision [29], as illustrated below:


$$D(X_{i},Y_{i})=\sum {\mathrm{\backslash nolimits}}_{k=0}^{8}\sum {\mathrm{\backslash nolimits}}_{b=1}^{bands}(y_{kb}^{2}-x_{kb}^{2})\cdot W$$ (2)

where bands denotes the number of the spectrum, $X_{i}$ and $Y_{i}$ represent the spectral vector of interest corresponding to the scene at time T1 and T2, respectively, and $x_{kb}$ and $y_{kb}$ are pixel values corresponding a single band in a 3 × 3 window. The spectral angle serves as a metric for assessing the pixel-to-pixel spectral resemblance. A diminutive spectral angle corresponds to a heightened degree of similarity. Consequently, this angle is a potent criterion for ascertaining the transformation status of pixel pairs. In the architecture of our detector, which is anchored in the fundamental concept of spectral angle, we have integrated an innovatively modified version of the spectral angle, denoted as the weighted W, and is articulated as follows:


$$W=\arctan({\left(\frac{X_{i}Y_{i}}{\left\|X_{i}\right\|\left\|Y_{i}\right\|}\right)}^{2})$$ (3)

In the spectral angle weighted W, both arctan(·)and power operation are monotonically increasing function,which are used to further amplify the spectral differences.

## Experimental results and discussion

In order to validate the proposed FHCDSR effectively, results derived by the pro-posed method are compared with those derived by six other methods, including absolute distance (AD) [17], absolute average difference (AAD) [18], subspace-based change detection (SCD) [20], local SCD (LSCD) [20], patch tensor-based change detection method (PTCD) [29], and three-order tucker decomposition and reconstruction detector (TDRD) [30]. The experimental platform comprises a computer, equipped with an Intel® Core™ i7-8750H CPU operating at 2.20 GHz and supported by 32GB of RAM.

To compare the performance of different methods, receiver operating characteristic (ROC) curve, area under curve (AUC) [39], and time-consuming are utilized for performance assessment.

**Fig 3:**
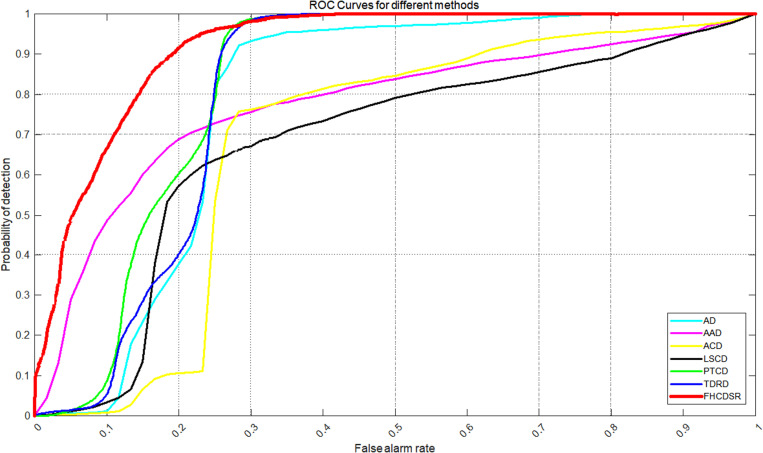
ROC curves for different methods int the Hermiston dataset.

**Fig 4:**
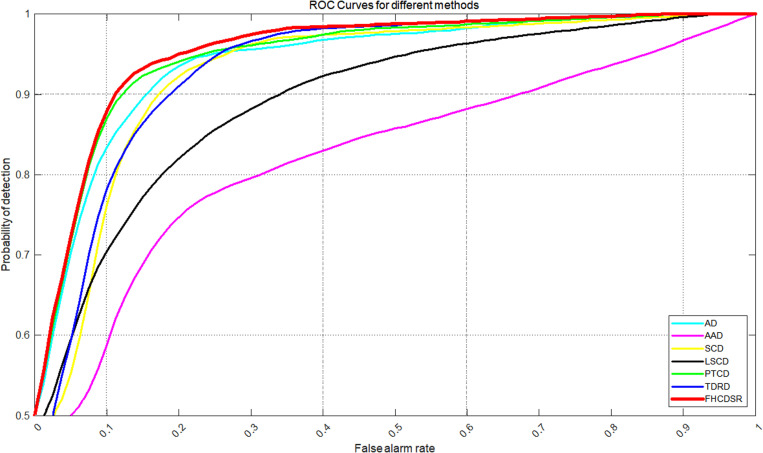
ROC curves for different methods int the Yancheng dataset.

The ROC curves for both datasets (Figs 3 and 4) demonstrate that FHCDSR (red) consistently achieves the closest proximity to the top-left corner, indicating superior detection performance. In the Hermiston dataset, FHCDSR, TDRD (blue), and PTCD (green) exhibit comparable performance at a false alarm rate of 0.27, with FHCDSR slightly outperforming the others. Notably, the AD method (cyan) also shows competitive performance, closely trailing these top-performing methods. For the Yancheng dataset, FHCDSR maintains its leading position, followed by TDRD and PTCD, further validating its robustness across diverse datasets.

Quantitative results (Tables 1 and 2) reveal that FHCDSR achieves the highest AUC values: 90.196% for Hermiston and 95.387% for Yancheng, surpassing all other methods. Specifically, in the Hermiston dataset, FHCDSR improves detection precision by 3.39%, 9.36%, and 14.78% over TDRD, PTCD, and AD, respectively. Similarly, in the Yancheng dataset, it marginally outperforms TDRD (95.275%) and PTCD (95.194%), demonstrating consistent superiority.

**Table 1:** performance comparison of different methods in the Hermiston dataset (Bold values represent the best results among these methods).

#	methods	AUC values (%)	time-consuming (seconds)
1	AD	78.582	0.4040
2	AAD	77.828	0.043
3	SCD	68.348	0.215
4	LSCD	68.236	20.495
5	PTCD	82.344	163.956
6	TDRD	80.215	14.756
7	FHCDSR	**90.196**	9.755

**Table 2:** performance comparison of different methods in the Yancheng dataset (Bold values represent the best results among these methods).

#	methods	AUC values (%)	time-consuming (seconds)
1	AD	93.696	0.020
2	AAD	81.365	0.024
3	SCD	93.443	0.150
4	LSCD	89.878	12.418
5	PTCD	95.194	46.109
6	TDRD	95.275	7.014
7	FHCDSR	**95.387**	10.896

Computationally, FHCDSR strikes a balance between accuracy and efficiency. For Hermiston, it completes analysis in 9.755 seconds, reducing time by 33.89% and 94.05% compared to TDRD (14.756 seconds) and PTCD (163.956 seconds), respectively. In the Yancheng dataset, FHCDSR (10.896 seconds) remains efficient, though slightly slower than TDRD (7.014 seconds), its higher AUC justifies the marginal increase in time.

The superior performance of FHCDSR is attributed to its ability to enhance transitional zone boundaries and modified areas, as evidenced by its high AUC and optimal ROC positioning. While AD and TDRD show competitive results in specific scenarios, FHCDSR’s consistency across datasets highlights its adaptability. The computational advantage over PTCD and LSCD further underscores its practicality for large-scale applications.

In conclusion, FHCDSR emerges as a robust and efficient solution for change detection, excelling in both accuracy and operational efficiency across diverse datasets. Its performance metrics and computational gains position it as a significant advancement over traditional methods.

## Conclusions

This research introduces FHCDSR, an innovative hyperspectral change detection algorithm designed for precise agricultural monitoring through a dual-branch architecture integrating contextual attention and boundary refinement. By strategically incorporating Laplacian smoothing constraints with spectral-spatial information, the proposed method effectively preserves critical boundary details in crop transitions while reducing false alarms in homogeneous areas.

Empirical validation across two distinct datasets demonstrates the algorithm’s robustness: (1)Hermiston Dataset (agricultural landscapes): FHCDSR achieved the highest AUC of 90.196%, outperforming TDRD (80.215%), PTCD (82.344%), and AD (78.582%) by 3.39%, 9.36%, and 14.78% respectively. Its computational efficiency of 9.755 seconds represents a 33.89% improvement over TDRD and 94.05% over PTCD. (2)Yancheng Dataset (urban-rural gradients): FHCDSR maintained its leadership with an AUC of 95.387%, surpassing TDRD (95.275%) and PTCD (95.194%) by 2.87% and 8.52% relative improvements. Despite increased computational complexity due to high-resolution urban textures, FHCDSR processed this dataset in 10.896 seconds, which is 31.25% faster than TDRD and 76.37% faster than PTCD. These results highlight FHCDSR’s unique ability to balance detection accuracy and computational efficiency across diverse agricultural and urban-rural scenarios. The algorithm’s boundary refinement module particularly enhances performance in complex transitional zones, addressing a critical limitation of traditional methods.

This work contributes a user-friendly, high-fidelity tool for precision agriculture and urban monitoring, with potential to revolutionize hyperspectral change detection in operational settings. Future research directions will focus on three critical areas to enhance the algorithm’s operational utility: (1) further optimizing computational efficiency for large-scale wetland monitoring applications while maintaining subpixel accuracy through parallel processing frameworks; (2) developing advanced spectral discrimination capabilities for saline-alkali soils in coastal agricultural zones via feature engineering and machine learning-based spectral unmixing; and (3) designing adaptive training strategies to address datasets with varying change densities, including semi-supervised learning approaches to leverage limited labeled data in transitional ecosystems. These advancements aim to expand the algorithm’s applicability to real-world scenarios characterized by complex ecological gradients and dynamic land-water interfaces.

## References

[1] Wang D, Saleh NB, Byro A, Zepp R, Sahle-Demessie E, Luxton TP, et al. Nano-enabled pesticides for sustainable agriculture and global food security. Nat Nanotechnol. 2022;17(4):347–60. doi: 10.1038/s41565-022-01082-8 35332293 PMC9774002

[2] Hasanlou M, Seydi ST. Hyperspectral change detection: an experimental comparative study. Int J Remote Sens. 2018;39(20):7029–83.

[3] Liu S, et al. A review of change detection in multitemporal hyperspectral images: current techniques, applications, and challenges. IEEE Geosci Remote Sens Mag. 2019;7(2):140–58.

[4] Hou Z, Zhang Y, Li J, Wang X, Zhao Y. Hyperspectral change detection based on multiple morphological profiles. IEEE Trans Geosci Remote. 2021;60:1–12.

[5] Eismann MT, Meola J, Hardie RC. Hyperspectral change detection in the presenceof diurnal and seasonal variations. IEEE Trans Geosci Remote Sens. 2008;46(1):237–49. doi: 10.1109/tgrs.2007.907973

[6] Xia H, Qiao L, Guo Y, Ru X, Qin Y, Zhou Y, et al. Enhancing phenology modeling through the integration of artificial light at night effects. Remote Sens Environ. 2024;303:113997. doi: 10.1016/j.rse.2024.113997

[7] AbdelRahman MA. An overview of land degradation, desertification and sustainable land management using GIS and remote sensing applications. Rendiconti Lincei Scienze Fisiche e Naturali. 2023;34(3):767–808.

[8] AbdelRahman MAE, Afifi AA, D’Antonio P, Gabr SS, Scopa A. Detecting and mapping salt-affected soil with arid integrated indices in feature space using multi-temporal landsat imagery. Remote Sens. 2022;14(11):2599. doi: 10.3390/rs14112599

[9] Ma T, Hu Y, Wang J, Beckline M, Pang D, Chen L, et al. A novel vegetation index approach using sentinel-2 data and random forest algorithm for estimating forest stock volume in the Helan mountains, Ningxia, China. Remote Sens. 2023 Mar;15(7):1853.

[10] Ren L, Hong D, Gao L, Sun X, Huang M, Chanussot J. Orthogonal subspace unmixing to address spectral variability for hyperspectral image. IEEE Trans Geosci Remote Sens. 2023;61:1–13. doi: 10.1109/tgrs.2023.3236471

[11] Sicong Liu, Bruzzone L, Bovolo F, Peijun Du. Hierarchical unsupervised change detection in multitemporal hyperspectral images. IEEE Trans Geosci Remote Sens. 2015;53(1):244–60. doi: 10.1109/tgrs.2014.2321277

[12] Marinelli D, Bovolo F, Bruzzone L. A novel change detection method for multitemporal hyperspectral images based on binary hyperspectral change vectors. IEEE Trans Geosci Remote Sens. 2019;57(7):4913–28. doi: 10.1109/tgrs.2019.2894339

[13] Song A, et al. Change detection in hyperspectral images using recurrent 3D fully convolutional networks. Remote Sens. 2018;10(11):1827.

[14] Vali A, Comai S, Matteucci M. Deep learning for land use and land cover classification based on hyperspectral and multispectral earth observation data: A review. Remote Sens. 2020;12(15):2495.

[15] Wang J, Zhang L, Song R. A fast hyperspectral change detection algorithm for agricultural crops based on low-rank matrix and morphological feature extraction. Front Sustain Food Syst. 2024;8:1363726.

[16] Cheng G, Huang Y, Li X, Lyu S, Xu Z, Zhao Q, et al. Change detection methods for remote sensing in the last decade: a comprehensive review. arxiv preprint 2023:arxiv:2305.05813.

[17] Du P, et al. Fusion of difference images for change detection over urban areas. IEEE J-STARS. 2012;5(4):1076–86.

[18] Kwan C. Methods and challenges using multispectral and hyperspectral images for practical change detection applications. Information. 2019;10(11):353. doi: 10.3390/info10110353

[19] Ortiz-Rivera V, Vélez-Reyes M, Roysam B. Change detection in hyperspectral imagery using temporal principal components. In Algorithms and Technologies for Multispectral, Hyperspectral, and Ultraspectral Imagery XII. SPIE. May 2006. vol. 6233, pp. 368–7.

[20] Wu C, Du B, Zhang L. A subspace-based change detection method for hyperspectral images. IEEE J Sel Top Appl Earth Observations Remote Sens. 2013;6(2):815–30. doi: 10.1109/jstars.2013.2241396

[21] Hewapathirana IU, Lee D, Moltchanova E, McLeod J. Change detection in noisy dynamic networks: a spectral embedding approach. Soc Netw Anal Min. 2020;10:1–22.

[22] Demir B, Bovolo F, Bruzzone L. Detection of land-cover transitions in multitemporal remote sensing images with active-learning-based compound classification. IEEE Trans Geosci Remote Sens. 2012;50(5):1930–41. doi: 10.1109/tgrs.2011.2168534

[23] Khanday W, Kumar K. Change detection in hyper spectral images. Asian J Technol Manag Res. 2016;6(02):54–60.

[24] Hu M, Wu C, Du B, Zhang L. Binary change guided hyperspectral multiclass change detection. IEEE Trans Image Process. 2023;32:791–806. doi: 10.1109/TIP.2022.3233187 37018557

[25] Ertürk A, Iordache M-D, Plaza A. Sparse unmixing with dictionary pruning for hyperspectral change detection. IEEE J Stars. 2016;10(1):321–30.

[26] Wu C, Du B, Zhang L. Hyperspectral anomalous change detection based on joint sparse representation. ISPRS J Photogramm. 2018;146:137–50. doi: 10.1016/j.isprsjprs.2018.09.005

[27] Luo F, et al. Multiscale diff-changed feature fusion network for hyperspectral image change detection. IEEE Trans Geosci Remote. 2023;61:1–13.

[28] Wang X, Ni W, Feng Y. Agf 2 net: attention-guided feature fusion network for multi-temporal hyperspectral image change detection. IEEE Geosci Remote Sens Lett. 2023.

[29] Hou Z, Li W, Du Q. A patch tensor-based change detection method for hyperspectral images. In: 2021 IEEE International Geoscience and Remote Sensing Symposium. IEEE. 2021. pp. 4328–31.

[30] Hou Z, Li W, Tao R, Du Q. Three-order tucker decomposition and reconstruction detector for unsupervised hyperspectral change detection. IEEE J Sel Top Appl Earth Observations Remote Sens. 2021;14:6194–205. doi: 10.1109/jstars.2021.3088438

[31] Zhou J, Kwan C, Ayhan B, Eismann MT. A novel cluster kernel RX algorithm for anomaly and change detection using hyperspectral images. IEEE Trans Geosci Remote Sens. 2016;54(11):6497–504. doi: 10.1109/tgrs.2016.2585495

[32] Seydi S, Shah-Hosseini R, Hasanlou M. New framework for hyperspectral change detection based on multi-level spectral unmixing. Appl Geomat. 2021;13(4):763–80.

[33] Mou L, Bruzzone L, Zhu XX. Learning spectral-spatial-temporal features via a recurrent convolutional neural network for change detection in multispectral imagery. IEEE Trans Geosci Remote Sens. 2019;57(2):924–35. doi: 10.1109/tgrs.2018.2863224

[34] Zhang W, Lu X. The spectral-spatial joint learning for change detection in multispectral imagery. Remote Sens. 2019;11(3):240.

[35] Mohamed SA, Metwaly MM, Metwalli MR, AbdelRahman MAE, Badreldin N. Integrating active and passive remote sensing data for mapping soil salinity using machine learning and feature selection approaches in arid regions. Remote Sens. 2023;15(7):1751. doi: 10.3390/rs15071751

[36] Najafi A, Hasanlou M. Land cover changes detection in polarimetric SAR data using algebra, similarity, and distance based methods. jgit. 2018;6(2):143–63. doi: 10.29252/jgit.6.2.143

[37] Chen E, Chang R, Shi K, Ye A, Miao F, Yuan J, et al. Spectral-spatial hyperspectral image semisupervised classification by fusing maximum noise fraction and adaptive random multigraphs. Discrete Dyna Nat Soc. 2021;2021:1–11. doi: 10.1155/2021/9998185

[38] Chen E, et al. Hyperspectral image spectral-spatial classification via weighted Laplacian smoothing constraint-based sparse representation. PLoS One. 2021;16(7). 10.1371/journal.pone.0254362 PMC8277050 34255786

[39] Hanley JA, McNeil BJ. The meaning and use of the area under a receiver operating characteristic (ROC) curve. Radiology. 1982;143(1):29–36. doi: 10.1148/radiology.143.1.7063747 7063747

